# Variability of Glycemic Outcomes and Insulin Requirements Throughout the Menstrual Cycle: A Qualitative Study on Women With Type 1 Diabetes Using an Open-Source Automated Insulin Delivery System

**DOI:** 10.1177/19322968221080199

**Published:** 2022-03-07

**Authors:** Darius Mewes, Mandy Wäldchen, Christine Knoll, Klemens Raile, Katarina Braune

**Affiliations:** 1 Department of Pediatric Endocrinology and Diabetes, Charité—Universitätsmedizin Berlin, Berlin, Germany; 2 School of Sociology, University College Dublin, Dublin, Ireland; 3 Berlin Institute of Health (BIH), Berlin, Germany; 4 Institute of Medical Informatics, Charité—Universitätsmedizin Berlin, Berlin, Germany

**Keywords:** glycemic variability, insulin sensitivity, sex hormones, menstrual cycle, automated insulin delivery, open-source

## Abstract

**Background:**

The impact of hormone dynamics throughout the menstrual cycle on insulin sensitivity represents a currently under-researched area. Despite therapeutic and technological advances, self-managing insulin therapy remains challenging for women with type 1 diabetes (T1D).

**Methods:**

To investigate perceived changes in glycemic levels and insulin requirements throughout the menstrual cycle and different phases of life, we performed semi-structured interviews with 12 women with T1D who are using personalized open-source automated insulin delivery (AID) systems. Transcripts were analyzed using thematic analysis with an inductive, hypothesis-generating approach.

**Results:**

Participants reported significant differences between the follicular phase, ovulation, and luteal phase of the menstrual cycle and also during puberty, pregnancy, and menopause. All participants reported increased comfort and safety since using AID, but were still required to manually adjust their therapy according to their cycle. A lack of information and awareness and limited guidance by health care providers were frequently mentioned. Although individual adjustment strategies exist, achieving optimum outcomes was still perceived as challenging.

**Conclusions:**

This study highlights that scientific evidence, therapeutic options, and professional guidance on female health-related aspects in T1D are insufficient to date. Further efforts are required to better inform people with T1D, as well as for health care professionals, researchers, medical device manufacturers, and regulatory bodies to better address female health needs in therapeutic advances.

## Introduction

Diabetes is one of the most common chronic conditions in women, and the global incidence of type 1 diabetes (T1D) and type 2 diabetes (T2D) has been on the rise for multiple decades.^1^ Recently, therapeutic and technological advances in diabetes care such as continuous glucose monitoring (CGM) systems and continuous subcutaneous insulin infusion (CSII) have facilitated the development of automated insulin delivery (AID) systems—also called “(Hybrid-)Closed-Loop Systems” or an “Artificial Pancreas.” The control algorithms used in AID systems automate and continuously adjust insulin dosage based on changes in glycemic levels and other factors such as carbohydrate intake. Randomized controlled trials and observational studies have supported the ability of these systems to improve glycemic outcomes, decrease hypoglycemic events, and improve quality of life in people with diabetes (PwD) of various age groups^2-4^ and in women with T1D during pregnancy.^5-7^

Prior to the availability of commercially developed AID systems, a community of people affected by T1D behind the hashtag #WeAreNotWaiting have collaboratively developed open-source AID algorithms and openly shared their source code and documentation online. In these systems, existing medical devices are connected with an app running an open-source control algorithm on their smartphones (AndroidAPS for Android phones, Loop for Apple iPhones) or on a small microcontroller (OpenAPS). Worldwide, an estimated number of several thousand PwD^8,9^ are currently using open-source AID, of which approximately 44% are women.^9-11^ Observational studies^9,11,12^ have shown safety and efficacy for open-source AID for PwD of various age groups and genders alike. User experiences reflect quality-of-life improvements and describe the customizability and range of personalized features of these systems as important characteristics.^13-17^

For women living with T1D, managing diabetes can be particularly challenging throughout different phases of life. Several studies have shown that women with T1D and T2D are less likely to reach targets in hemoglobin A1c, blood pressure, and low-density lipoprotein cholesterol as recommended by therapeutic guidelines,^18^ compared with men, with possible explanations for these disparities remaining unclear.^19,20^

The impact of sex hormone dynamics on insulin sensitivity and glucose metabolism is subject of constant scientific debate.^21-23^ Variations in insulin sensitivity throughout the menstrual cycle have been previously studied in women without diabetes. However, the underlying molecular mechanisms are complex, and variable correlations of female sex hormones and insulin sensitivity were observed. Several studies that examined intravenous glucose tolerance in smaller cohorts found either increased insulin resistance^24-26^ or no significant differences in insulin sensitivity^27^ during the luteal phase of the menstrual cycle. A euglycemic, hyperinsulinemic clamp study found no insulin sensitivity differences in relation to menstrual cycle phases.^28^ A longitudinal study that investigated fasting glucose and insulin concentrations in a larger cohort of 257 women without diabetes showed significant changes in insulin resistance associated with estradiol and progesterone concentrations and higher insulin resistance during the luteal phase,^22^ in line with observational studies that found significant correlations of estradiol concentrations in saliva and insulin levels in 204 women regardless of their current menstrual cycle phase,^29^ and between estradiol and insulin concentration in 845 postmenopausal women.^30^

Despite the available evidence on the influence of sex hormones on glycemic levels in individuals without diabetes, research on women with diabetes is sparse but equally controversial. First observations suggesting an association of diabetes and the menstrual cycle were made early in the history of insulin therapy in the 1940s, where cyclic changes in blood glucose concentrations were observed in seven girls with T1D prior to their menarche.^31^ Further studies from the 1990s and the early 2000s have found menstrual irregularities to occur more frequently in adolescents^32^ and adults with T1D.^33,34^ Insulin sensitivity in relation to the menstrual cycle was first investigated by hyperglycemic, hyperinsulinemic clamp studies in the 1990s. A clamp study of 16 women reported marked heterogeneity in glucose metabolism in all and lower insulin sensitivity during the luteal phase in some of the participants.^35^ These findings could not be confirmed by others.^36,37^ A population-based study from 1996 on 124 women with T1D first highlighted self-reported changes in glycemic levels around menstruation in 61% of the participants.^38^ Data throughout several complete menstrual cycles were first assessed in 2004 by a pilot study of four women with T1D using CGM,^39^ where different interindividual sensor glucose patterns were found; however, these patterns were consistent over several cycles of the same person.^39^ An observational study of 12 women using CSII and CGM combined found hyperglycemia to occur more frequently around ovulation and the early luteal phase compared with the early follicular phase.^40^ Controversially, a recent study of seven participants found postexercise hyperglycemia to be more prominent during the follicular phase.^41^

Despite these implications, sex hormone–related aspects are—except for pregnant women with diabetes^42^—not sufficiently considered in therapeutic guidelines, medical device development, and clinical trials to date. The use of AID systems, and customizable open-source AID systems in particular, could facilitate the investigation of insulin needs and glycemic patterns in relation to the menstrual cycle, and thus contribute to the evidence base of this under-researched area. Therefore, this explorative study aimed to investigate user experiences of women living with T1D and using open-source AID systems in relation to their menstrual cycles, thereby leveraging experienced-based evidence and ideas for further improvement of AID systems from the #WeAreNotWaiting community and enabling further research in the field of T1D and women’s health.

## Methods

### Study Design

As part of the patient-led OPEN project,^43^ a questionnaire for the assessment of participant demographics and a schedule for semi-structured interviews were created by the OPEN team. Interview questions were designed based on previous reports of open-source AID users in response to the DIWHY survey,^14,44^ on discussions related to the study topic between open-source AID users in online peer-support groups of the #WeAreNotWaiting community and on the research team’s (KB, MW, KR) personal experience with T1D and using open-source AID. The interview framework was pilot-tested with two women using open-source AID before further participants were enrolled.

### Inclusion Criteria

Participants were eligible if they met the following inclusion criteria: >18 years of age, biological sex was female, living with T1D, using an open-source AID system for at least six months, and were proficient in either English or German at conversation level. No specific exclusion criteria applied.

### Recruitment

To specifically target open-source AID users of different ages and internationally, recruitment was carried out through social media. Announcements were posted both publicly (eg, on Twitter using the hashtag #WeAreNotWaiting) and in online peer-support groups for open-source AID (eg, the Facebook groups “Looped,” approximately 23 000 members, and “Looped-DE,” approximately 2000 members in July 2020). Participation was entirely voluntary with no financial compensation provided. The study was conducted according to the guidelines of the Declaration of Helsinki, and approved by the Institutional Review Board (or Ethics Committee) of Charité—Universitätsmedizin Berlin (protocol code EA2/122/20, July 7, 2020). Prior to the interviews, participants were informed about the professional background and characteristics of the researchers performing the interviews and their interests and aims in pursuing this research. In addition, a detailed information sheet was provided to all participants and their electronic consent was obtained. Participants were recruited from July 2020 to January 2021. With a target sample size of 10 to 15, participants were purposively sampled until data saturation occurred.^45-49^

### Procedures

Semi-structured interviews with 12 participants were conducted via secure online video calls in either German or English. Online interviews were conducted by DM and CK with the use of encrypted online video chat services Zoom (Zoom Video Communications, San Jose, California) and Google Meet (Google Inc., Mountain View, California). The calls lasted 45 to 60 minutes each. The questionnaire assessing demographics and personal female health and diabetes-related history (Supplemental Material) was sent to the participants prior to the video call. During the interviews, the researcher asked the participants to share their observations and perceived challenges related to their diabetes during different phases of life (eg, puberty, menopause, pregnancy) and throughout the menstrual cycle (eg, if they had noticed variability in glycemic outcomes and an estimate of the relative changes in overall insulin requirements throughout the menstrual cycle in percentage). Next, the interviewer assessed participants’ individual solution strategies and manual “workarounds” with their open-source AID system and otherwise to address these challenges. Interviews finished with discussions on users’ ideas of how to better automate control algorithms and further improve future generations of AID systems for women.

### Data Collection and Analysis

Data collection was carried out in accordance with national data protection regulations. The interviews were audio and video recorded, transcribed, and de-identified. Transcribed texts were imported into the MAXQDA Plus 2020 software (VERBI GmbH Berlin, Germany).^50^ Given the scarcity of existing qualitative research on the topic of women’s health and T1D, an explorative and inductive approach was chosen to generate new hypotheses and remain open to unexpected findings. The analysis and generation of themes were carried out by the research team collaboratively (DM, MW, CK, KB). It should be noted that “themes” refer to interpretive stories about particular patterns of shared meaning in the data. These were developed in interaction with the researchers’ theoretical assumptions, their analytic skill, and the collected data. Thematic analysis was used to analyze the data, including data familiarization, coding, generation of themes, theme review, theme definition, and naming.^50^ The thematic analysis did not strictly follow procedures such as coding or achieving inter-rater reliability between researchers, and instead enabled reflection and engagement by the researchers throughout the analytic process.^50^ Iterative discussion rounds were held until consensus between researchers was achieved. The COREQ (COnsolidated criteria for REporting Qualitative research) checklist was used to guide reporting.^51^

## Results

Of the 28 women who expressed their interest in participating, 12 participants based in four different countries were recruited, meeting our target sample with no dropouts. Participants had a median age of 39 years, ranging from 24 to 56 years, and a median experience of using an open-source AID system (OpenAPS, Loop, or AndroidAPS) of 21 months, ranging from 12 to 48 months. Further demographics and health characteristics are presented in Table 1. Content analysis of the data provided six themes with several subthemes, as presented in Table 2.

**Table 1.** Participant Demographics, Diabetes-Related and Gynecological History.

No.	Age (years)	Country of residence	AID system(s)	AID experience (mo)	No. of pregnancies	Contraceptive method(s)	Mean cycle length (d)	Years since T1D diagnosis (y)
1	56	Germany	AndroidAPS, OpenAPS	48	2	Nonhormonal	In menopause	21
2	31	Germany	AndroidAPS, Loop	31	None	Barrier and sympto-thermal methods	34	19
3	33	Germany	AndroidAPS	13	2	Hormonal IUD	26	28
4	46	Germany	AndroidAPS	25	1	None	28	29
5	26	United States	Loop	31	None	Hormonal IUD	Not menstruating	24
6	49	Germany	AndroidAPS	16	3	Barrier methods	29	27
7	47	Australia	AndroidAPS, Loop	22	None	Hormonal IUD	In menopause	42
8	31	United States	Loop	20	1	Copper IUD	36	20
9	26	Germany	AndroidAPS	16	None	Barrier methods	30	23
10	24	Germany	AndroidAPS	12	None	Nonhormonal	29	17
11	45	France	Loop	17	2	None	26	23
12	52	Germany	AndroidAPS	31	4	Barrier methods	In menopause	43

Abbreviations: AID, automated insulin delivery; IUD: intrauterine device; T1D, type 1 diabetes.

**Table 2.** Content Analysis: Theme Structure, Definition, Example Quotes, and Respondent Profiles.

Theme	Definition	Example quote(s)	Respondent profile
(A) Improvements through open-source AID			
(A1) Increased quality of life	Refers to perceived improvements in everyday life and reduced diabetes-related distress following the implementation of open-source AID	“This is the easiest and safest my care has ever been.”	26-year-old American woman, using Loop for 2.5 years
(A2) Improved clinical outcomes	Refers to the perceived changes in clinical outcomes (eg, fewer hypoglycemia and hyperglycemia, more time-in-range) and perceived increases in safety following the implementation of open-source AID	“I can tell: June 13th, 2018. First time I slept through the first night, with Loop. [^Previously^,] I was [. . .] very often woken up either by my own hypoglycemia, by noticing [the symptoms] myself, or by an alarm. The loop has made it: It was really the first night I didn’t wake up to some stupid alarms. [. . .] And I am no longer afraid that it will happen. Because I know someone will take care of it. My app.”	52-year-old German woman, using AndroidAPS for 2.5 years
(B) Variations in glycemia and insulin requirements			
(B1) Intraindividual differences	Refers to variability in glycemic outcomes and insulin requirements throughout the menstrual cycle and different life stages observed by the women	“It’s like a major frustration for me because the first couple weeks of my cycle are so nice and then the last half is kind of a disaster zone.”	31-year-old American woman, using Loop for 1.5 years
(C) Additional effort to achieve therapy outcomes			
(C1) Gender inequality	Refers to the perceived differences between men and women in therapy effort needed to achieve the desired outcomes	“I always find it so inequitable when men [. . .] brag about their great blood sugar levels. I would wish them a month in the life of a woman and then see how they deal with it. That is, I would wish them humility. I think if you never experienced it yourself, you can’t imagine what it’s like.”	49-year-old German woman, using AndroidAPS for 16 months
(C2) Causing distress	Refers to the additional burden perceived by the women related to female health–related challenges in managing diabetes	“You know, you can do your best, but it won’t be good enough.”	31-year-old American woman, using Loop for 1.5 years
(D) Limited awareness and support			
(D1) Limited awareness pre-AID	Refers to the novelty of the observations since using open-source AID and the participants’ unawareness of a possible correlation between menstrual cycle and diabetes prior to using an AID system	“I notice the [correlation] very prominently. I also noticed it with MDI, but I could not attribute it that way.” “I would say that you can see a tendency that in the second half of the cycle the levels and the insulin requirements are higher. Before Loop, I didn’t even notice anything. With the closed-loop, you notice it way more when something is off.”	56-year-old German woman, using OpenAPS and AndroidAPS for four years 31-year-old German woman, using Loop for 2.5 years
(D2) Limited HCP support	Refers to therapy adjustments that were often attempted “trial and error” with limited professional medical guidance and the perceived lack of awareness toward sex- and gender-specific aspects in diabetes care among physicians and other HCPs, in the fields of both endocrinology/diabetes care and obstetrics/gynecology	“At a time when it would have been very important for me, for example, when I had children, my endocrinologist never pointed out to me that we had to adjust anything. I’ve only just noticed now that there is a women’s area in the Loop [groups]. Makes sense, that [the therapy] is specifically adjusted during pregnancy. But to take a closer look at the cycle? [. . . ] No one said ‘[. . .] please increase your basal rates every 26 days.’ That was just not a discussion to have with the endocrinologist. [. . .] Now I am with a woman [endocrinologist] and I asked her about menopause. She said yes, she didn’t know either, she’d have to read up on it. [. . .] I asked her: ‘How is it now with [diabetes], menopause, what to expect?’ And then she started [telling me] about night sweats. And I said, ‘No, that’s not the question at all. I would like to know how the blood glucose reacts to it?’ And then she did not know the answer.”	52-year-old German woman, using AndroidAPS for 2.5 years
(E) Solution strategies			
(E1) Peer-support	Refers to support provided by other people with diabetes, often online in social media groups	“I am on a lot of Facebook groups including a ‘Looping in Pregnancy’ one and a ‘Breastfeeding and Type 1 Diabetes’ one. Those are particular groups I’d be sooner to ask sort of women-specific or hormonal questions on.”	31-year-old American woman, using Loop for 1.5 years
(E2) Cycle documentation	Refers to ways of documenting the menstrual cycle	“I used to document the first day of my period in my phone. And in my paper calendar. Then I have discovered the insulin age [field] in AndroidAPS which I am using now. Practically I do not enter my insulin age in the app, but [use if for] the first day of my period. [. . .] That is actually perfectly suited to get a bit of an overview, how far along [in the cycle] I am right now.”	49-year-old German woman, using AndroidAPS for one year
(E3) Open-source AID features	Refers to the use of already existing features in open-source AID (eg temporary overrides, profile switches)	“When I encounter higher levels, I switch to a temporary override relatively quickly. [. . .] You can set [all parameters] to 110%, 120% and so on.”	31-year-old German woman, using Loop for 2.5 years
(E4) Fake carbs	Refers to carbohydrate entries in the AID system without actually consuming them	“I tried changing my profile, it really means everything ISF and everything changed and that did not work. [Now I am] only changing the basal rate and then possibly correct again if I notice it is not working. Then I add a few more fake carbs. [. . .] The Loop thinks I still have carbohydrates [on board], but there aren’t any. And [. . .] then, the Loop reacts a little more aggressively.”	46-year-old German woman, using AndroidAPS for two years
(E5) Manual changes of settings	Refers to manual adjustments of AID settings (eg, ISF, carb ratio, targets)	“The ISF. It’s not the case for older big ladies, like really big ladies. I think because they still have a lot of estrogen anyway because they are big. But for average size ladies, and there are some quite thin ladies I know, they are very very sensitive using ISFs of nine, ten, ten and a half. Which is about what toddlers use. And I did not change my ISF from five until I talked to some of these ladies and they’re like: ‘Yeah, I’ve had to put mine up to nine.’ And so, I tried that, and I got a flat line.”	47-year-old Australian woman, using Loop and AndroidAPS for 1.5 years
(E6) Exercise	Refers to intentional exercise in phases with high insulin resistance	“Before my period, for at least a week, I need a lot more insulin, so my insulin sensitivity is a lot lower. [. . .] I guess I exercise on purpose just to try to not increase the insulin by so much.”	31-year-old American woman, using Loop for 1.5 years
(F) Ideas for further improvements			
(F1) Further research and education	Refers to the perceived scarcity of the available literature and education on the topic of female health and diabetes	“I think we absolutely all need to learn about this. It’s probably only been in the last five or six, maybe ten, years that women had the chance to reflect on continuous glucose monitoring during their cycles. Before that it was just, you know, whatever. And I think also we—Because, I mean, everything is tested on men, generally white men, we do miss out on a lot of research. And this stuff is so important. I think this is something little girls need to know about. It’s not just the birds and the bees, it’s: ‘Hey, your insulin is going to need to do some weird stuff. It’s all going to be different’. Because none of us had any idea, right? Just wasn’t talked about. Wasn’t a thing.”	47-year-old Australian woman, using Loop and AndroidAPS for 1.5 years
(F2) Menstrual cycle-related automation	Refers to the ideas and suggestions for further automation and additional features to better cater to the users’ unmet needs	“It would help anticipate a little bit more in terms of, you know, I’m on day 20 and so this is where things are starting to be a little more resistant, but I don’t realize that. [. . .] Loop already talks to Apple Health, and I use the Apple Health app to track my cycle, so it doesn’t seem very far to take that information from Apple Health. [. . .] If Loop could already take into account when was the cycle ‘day one’ it would probably be helpful already.”	45-year-old French woman, using AndroidAPS for 1.5 years
(F3) Machine learning	Refers to the implementation of self-learning algorithms based on user data	“If it was learning from the data—I love the idea of Autotune but I don’t think it’s necessarily accurate for Loop specifically—if there was something like ‘I’ve noticed that it seems you really need, [. . .] my need seems to ramp up over time and then ramp down as opposed to being from day to day normal and then all of the sudden 20% more. [. . .] Your period is predicted to start in 12 days so I’m going to go up by 5 percent. And now 10. And now 15’. [. . .] If [the algorithm] learned based on experience—you know: ‘Your last three cycles your insulin needs were like this so I’m going to mimic that’.”	31-year-old American woman, using Loop for 1.5 years

Abbreviations: AID, automated insulin delivery; HCP, heath care professional; ISF, insulin sensitivity factor; MDI, multiple daily injections.

### Theme A: Improvements Through Open-Source AID

All participants expressed high satisfaction with open-source AID as their treatment option of choice, noting that it increased their quality of life (subtheme A1). One participant called it a “huge relief for life in comparison to the past” (33-year-old German woman, using AndroidAPS for one year); another described it as “the easiest and safest my care has ever been” (26-year-old American woman, using Loop for 2.5 years).

Improved clinical outcomes were also reported (subtheme A2). This was mostly associated with the availability of fast, predictive, and automated dosing of.

correction insulin in response to changes in sensor glucose, which to a large degree did not require frequent manual intervention. Decreases in hemoglobin A1c, increases in time-in-range, and fewer hypoglycemic events, especially at nighttime, were described frequently:

I can tell: June 13th, 2018. First time I slept through the first night, with Loop. [Previously], I was [. . . ] very often woken up either by my own hypoglycemia, by noticing [the symptoms] myself, or by an alarm. The loop has made it: It was really the first night I didn’t wake up to some stupid alarms. [. . .] And I am no longer afraid that it will happen. Because I know someone will take care of it. My app. (52-year-old German woman, using AndroidAPS for 2.5 years)

### Theme B: Variations in Glycemia and Insulin Requirements

**Figure 1.**
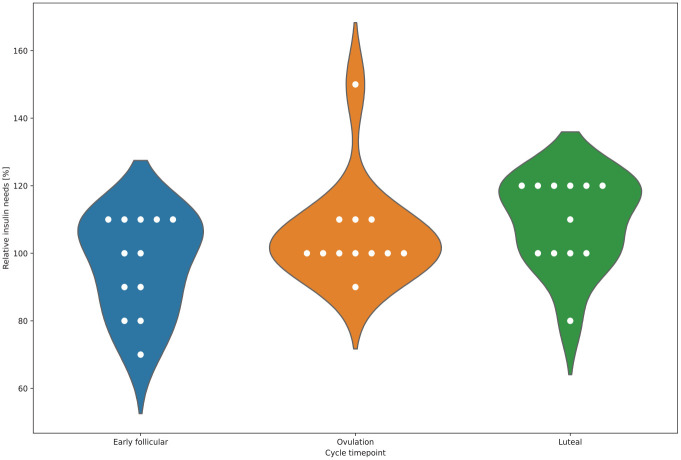
Relative changes (%) in self-reported insulin requirements of female open-source AID users during different menstrual cycle phases (blue: early follicular phase, orange: around ovulation, green: luteal phase). Abbreviation: AID, automated insulin delivery.

All participants reported having experienced changes in glycemic levels and insulin requirements associated with different phases of their menstrual cycle (subtheme B1), which required most of them (n = 10) to adjust their insulin therapy. Most participants (n = 10) reported experiencing regular fluctuations in glucose levels and insulin needs throughout the menstrual cycle, requiring them to change their settings. An overview of the interindividual differences throughout the follicular phase, around ovulation, and the luteal phase is summarized in Figure 1 and Table 3 based on the data reported by the participants (Supplementary Table 1).

**Table 3.** Self-Reported Perceived Changes in Glycemic Levels and Insulin Needs of Open-Source AID Users Throughout Different Phases of the Menstrual Cycle and Special Situations (Pregnancy, Menopause).

Menstrual cycle phase or special situation	Perceived changes in insulin needs
Follicular phase	With the onset of menses and the following two to three days, some (n = 5) women reported a sudden increase in insulin requirements and therefore the necessity to decrease their insulin delivery by 10% to 30%, while some (n = 4) needed to increase their dose by 10% to 20%. One woman reported a small decrease in insulin needs but does not regularly adapt settings accordingly. During menses, correlations between changing insulin needs and the occurrence and intensity of menstrual pain and other related symptoms, level of physical activity, and comorbidities were suspected. The late follicular phase up to the suspected day of ovulation was considered as the most “stable” and “easy to manage” in relation to glycemic levels. Insulin needs during that phase were considered as “normal” or “average.”
Around ovulation	Participants identified their ovulation to take place between cycle day 13 and day 21, depending on their cycle length. Some either reported to perceive specific physical symptoms (n = 6) such as one-sided abdominal pain or a “pulling sensation”, and increased libido, or used menstrual cycle tracking apps to identify their fertile window. A sudden increase in insulin requirements on ovulation day and the following one to two days of the cycle was reported by three participants, whereas one woman explained to experience decreased insulin needs, which has been more prominent after her first pregnancy but has become less noticeable since then.
Luteal phase	Post-ovulation, insulin needs were reported to be increased by up to 35% until the next cycle. Participants performed several different therapy adjustment strategies: two of the three women who experienced higher insulin needs during ovulation kept their more aggressive settings until the end of the cycle. One participant decreased her insulin “back to normal” (100%) temporarily after ovulation and then increased her insulin dose again for the last cycle week. One woman reported perceiving a small decrease in insulin demand for the last days of the cycle, whereas another woman explained being able to keep her settings on “default” (100%) throughout ovulation and until the next cycle begins. Of the five women who did not change settings during ovulation regularly, four reported to have a steady increase in insulin needs leading up to the next cycle. One woman reported being slightly more sensitive to insulin during that time and therefore decreased her intake to 80%.
Pregnancy	Different life phases and events, such as puberty, pregnancy, and menopause, were perceived as particularly challenging with respect to managing diabetes. Participants who had been pregnant in the past (n = 7) reported a constant effort of adapting their insulin requirements to the dynamic hormonal situation. Following pregnancies, participants reported that their insulin requirements needed to be reevaluated, rather than returning to their prepregnancy profiles. Furthermore, cycle length and strength of menstrual bleeding were perceived differently compared to before pregnancy.
Menopause	The transition into menopause was associated with decreased overall insulin requirements, changes in the length of the menstrual cycle, a decrease in menstrual bleeding, and ovulation frequency.

Abbreviation: AID, automated insulin delivery.

This was often associated with frustration:

It’s like a major frustration for me because the first couple weeks of my cycle are so nice and then the last half is kind of a disaster zone. (31-year-old American woman, using Loop for 1.5 years)

### Theme C: Additional Effort to Achieve Therapy Outcomes

All participants stated that even with using AID, manual therapy adjustments and “workarounds” related to the menstrual cycle were still necessary on a regular level, which caused them a constant time effort, cognitive load, and distress (subtheme C2), especially when compared with men (subtheme C1):

I always find it so inequitable when men [. . .] brag about their great blood sugar levels. I would wish them a month in the life of a woman and then see how they deal with it. That is, I would wish them humility. I think if you never experienced it yourself, you can’t imagine what it’s like. (49-year-old German woman, using AndroidAPS for 16 months)

Women of a younger age who managed multiple responsibilities such as work and childcare especially mentioned a lack of time to keep track of the changes and react to them. Even those participants with professional backgrounds in health care and personal interest in the topic of women’s health, as well as active members of the open-source online community who are in frequent exchange with other women with T1D, expressed that they rarely felt they were “in control” of their diabetes at all times. Even though the participants reported that switching to open-source AID had increased their knowledge about menstrual cycle effects on glucose levels and made diabetes management easier and safer, many expressed that certain challenges remain:

I’d say it’s still a major problem for me. I just remember being, just a few weeks ago, so frustrated. I just kept spiking high after meals, staying high overnight and stuff. So, I changed my settings and then in that instance, for whatever reason, I needed more than I thought, I guess. Or I would spike high after meals but then I would be low otherwise, so my basal was too strong but my carb ratio wasn’t good, or maybe I needed to pre-bolus longer than usual. (31-year-old American woman, using Loop for 1.5 years)

The frequent need for manual adjusting of settings and fine-tuning was also seen as straining:

Because anything that keeps me from having to constantly wonder about, you know: “Oh, okay. I’m getting a result I didn’t expect so now I have to do this whole troubleshooting in my head.” If the system just knew it’s the week before the period then that would save me some manual troubleshooting, I guess. (31-year-old American woman, using Loop for 1.5 years)

### Theme D: Limited Awareness and Support

The effect of the menstrual cycle on glucose levels and insulin requirements was first noticeably observed by the participants following the initiation of open-source AID use (subtheme D1):

I notice the [correlation] very prominently. I also noticed it with MDI, but I could not attribute it that way. (56-year-old German woman, using OpenAPS and AndroidAPS for four years)

Furthermore, support and awareness of women’s health and diabetes from endocrinologists and obstetricians/gynecologists were perceived to be limited (subtheme D2) by all of the participating women:

But I had a conversation with him about what sort of problems I could expect for menopause. And he said “Oh, should be a breeze.” [. . .] Yeah, so they’re clueless. Completely clueless. (47-year-old Australian woman, using Loop and AndroidAPS for 1.5 years)

If valuable suggestions regarding insulin therapy were brought up by health care providers, they were highly appreciated:

Usually she at least comes up with one helpful thing each time I go. I do a lot of research myself, a lot of thinking and testing myself. For her to come up with any additional thoughts, I think is pretty good. (31-year-old American woman, using Loop for 1.5 years)

### Theme E: Solution Strategies

Peer-support (subtheme E1) via online communities such as the “Looped” Facebook groups was common among interviewees. As an example, group video calls for setting optimizations were mentioned, and one woman reported that a friend with T1D regularly reminded her to consider the current cycle phase in relation to glycemic outcomes outside target range.

Except for three women who used a hormonal intrauterine device (IUD), are in menopause, or both, all participants stated that they regularly document their cycle and associated symptoms (subtheme E2). Methods of cycle tracking included apps such as “Clue,” “myNFP,” “Mein Kalender Flo,” “Period Tracker,” or default calendar apps on Android and Apple smartphones. Some (n = 3) also used paper calendars. Documented attributes were the beginning of menses, duration, intensity of bleeding, and suspected or calculated day of ovulation, and the fertile window. One woman explained how she used the “insulin age” field of AndroidAPS to document her cycle. She expressed not necessarily needing this field for its intended purpose as she generally replaces her insulin every few days. Instead, having her cycle documented “at a glance” together with sensor glucose levels and insulin delivery was described as helpful.

A significant concern among participants was increasing their insulin delivery too early and provoking hypoglycemia in return, which a participant described as at “minimum annoying, maximum dangerous” (31-year-old American woman, using Loop for 1.5 years). Therefore, many described their management strategies as reactive rather than preventative, and changes were not being made until a significant upward trend in glucose levels was witnessed after a few days. The most commonly (*n* = 9) used features of open-source AID (subtheme E3) were “Override Presets” (in Loop) and “Profile Switches” (in AndroidAPS). Both features enable the user to automatically apply relative changes of all parameters affecting dosage calculation, including basal rate (BR), insulin sensitivity factor (ISF), and carb ratio (CR). Both AID systems allow for the saving and naming of profiles:

In Loop there’s the “Override Presets” so I’ll do one at 80 or 90 percent of total insulin needs, and I’ll just put it on until I eventually am running high because of the changes. But then I put on the 120 percent preset to increase my insulin. (26-year-old American woman, using Loop for 2.5 years)

Another mentioned strategy (n = 2) was the intentional overestimation of carbohydrate intake—referred to as “fake carbs” (subtheme E4)—before or between meals. The participants explained they used this method in addition to using override presets or profile switches as described above, if necessary. Some (n = 2) explained that a relative change of BR, ISF, and CR combined does not work sufficiently for them. Instead, through personal experience, they have found that manually changing the settings one by one (subtheme E5) gives them better results. Features such as “Autosens,” an algorithm in AndroidAPS that estimates insulin sensitivity based on the user’s glucose deviations,^52^ were used for fine-tuning their ISF. Other strategies unrelated to insulin delivery were performing exercise (subtheme E6) in phases with increased insulin resistance.

### Theme F: Ideas for Further Improvements

Several ideas on how to further improve diabetes management for women using an open-source AID system were shared. First, the scarcity of information and research in the field (subtheme F1) was acknowledged by many interviewees:

I think we absolutely all need to learn about this. It’s probably only been in the last five or six, maybe ten, years that women had the chance to reflect on continuous glucose monitoring during their cycles. Before that it was just, you know, whatever. And I think also we—Because, I mean, everything is tested on men, generally white men, we do miss out on a lot of research. And this stuff is so important. I think this is something little girls need to know about. It’s not just the birds and the bees, it’s: “Hey, your insulin is going to need to do some weird stuff. It’s all going to be different.” Because none of us had any idea, right? Just wasn’t talked about. Wasn’t a thing. (47-year-old Australian woman, using Loop and AndroidAPS for 1.5 years)

The required technical skills and levels of digital literacy required to set up and use open-source AID systems were also acknowledged (n = 3). Therefore, the desire to have a better understanding of the automated decisions, for example, rationales for temporary BR adjustments, was expressed.

Besides hardware improvements such as devices with louder alarms and smaller dosage settings for the insulin pump, all participants expressed that the linkage of AID to the phases of their menstrual cycle (subtheme F2) would be beneficial and already feasible. Suggestions included the option to specify insulin dosage settings for individual cycle phases (*n* = 7) and pattern recognition (*n* = 6) for personalized profiles, for example, by the combination of different information in Apple Health:

It would help anticipate a little bit more in terms of, you know, I’m on day 20 and so this is where things are starting to be a little more resistant, but I don’t realize that. [. . .] Loop already talks to Apple Health, and I use the Apple Health app to track my cycle, so it doesn’t seem very far to take that information from Apple Health. [. . .] If Loop could already take into account when was the cycle “day one” it would probably be helpful already. (45-year-old French woman, using AndroidAPS for 1.5 years)

The implementation of self-learning machine learning algorithms (subtheme F3) was also envisioned:

If it was learning from the data—I love the idea of Autotune but I don’t think it’s necessarily accurate for Loop specifically—if there was something like “I’ve noticed that it seems you really need, [. . .] my need seems to ramp up over time and then ramp down as opposed to being from day to day normal and then all of the sudden 20% more. [. . .] Your period is predicted to start in 12 days so I’m going to go up by 5 percent. And now 10. And now 15.” . . . If [the algorithm] learned based on experience—you know: “Your last three cycles your insulin needs were like this so I’m going to mimic that.” (31-year-old American woman, using Loop for 1.5 years)

In this context, concerns regarding the ability of algorithms to cater to individual constellations and needs were raised:

I think you would have to have an absolutely regular cycle. And certainly, I had that as a young woman. But teenagers aren’t necessarily regular. Menopausal ladies aren’t necessarily regular. In the middle women are often having babies and then breastfeeding and having their cycles when breastfeeding. So, there’s a lot, awful lot, of potential for irregularity which is normal. It’s not abnormal to be irregular. And I think for the very young women who are just starting their cycles, they’ve got all sorts of stuff going on and I think manual control of that would be better, maybe. But I can see maybe for a few people yes. At certain stages of their life. Nice regular cycles. A bit busy with work and things to stop and think about it. Yeah, maybe it could work. (47-year-old Australian woman, using Loop and AndroidAPS for 1.5 years)

## Discussion

This study reports that women with T1D using an open-source AID system perceived a significant impact of changes in insulin needs throughout their menstrual cycle and throughout different events and phases of life, such as puberty, pregnancy, and menopause. The influencing factors were mostly unknown to them before they started using open-source AID systems and caused them to perform several workarounds to manually adjust their therapy. Although using open-source AID had an overall positive effect on glycemic outcomes and quality of life, the requirement to respond to variability in insulin needs was perceived as an individual burden. Health care provider awareness and knowledge, as well as publicly available information on menstrual cycles and diabetes, were perceived as limited. Our findings provide valuable insights into the challenges women face in managing T1D throughout life and yield suggestions to further improve future generations of AID systems for women, contributing to gender equality and improved quality of care.

Although qualitative studies on lived experiences with AID systems among adults, teenagers,^10^ and younger children^53^ exist, this is the first qualitative study focusing specifically on women as a user group outside the context of pregnancy. The literature describes similar improvements of clinical and patient-reported outcomes for PwD of various ages and genders since commencing open-source AID.^9-11^,^17^ However, our findings suggest that women with T1D have to undertake extra efforts to achieve these results. These findings align with others that have highlighted where currently available commercial AID systems do not meet their users’ expectations and either terminate use or come up with unexpected solutions.^54^ Findings like these should be a call to action for academia, developers and manufacturers of diabetes technology to closely work together with PwD in their research and product development at an early level, to generate research questions that matter to them and improve the products’ usability and efficacy.

Our findings on self-reported variable insulin requirements in relation to the menstrual cycle are in-line with the few previous studies on women with T1D using therapies other than AID^40,55^ and mirror the correlation of increased insulin resistance in the luteal phase observed in women without diabetes.^22^,^24-26^ However, this is the first qualitative study to report how women’s health-related challenges were experienced by and reacted to by women with T1D. Furthermore, this is the first study to report on what strategies and “workarounds” AID users perform to respond to dynamic changes in insulin demands. Although it was already self-reported by women in the 1990s that most perceive differences in glycemia around their menstruation and some adjust their therapy,^38^ there is still no therapeutic guidance on the topic.

It is acknowledged that several strengths and limitations apply to our study. Of particular strength is the multinational character and wide age range despite the small sample size, and the stakeholder engagement strategy directly including the experience and ideas of people with T1D and the open-source AID community during the study design.

For the purpose of this study, ethics approval was only provided for adult participants. While we could identify similarities between participants of different ages, women under the age of 24, with a diabetes duration shorter than 17 years, women with less than 12 months experience in using open-source AID, and women using hormonal contraception methods with higher systemic impact than in hormonal IUDs, such as oral contraception or hormonal implants, are not represented in our study. The likelihood of selection bias when recruiting participants via social media further limits broad generalizations to all women using open-source AID. Further investigations on larger cohorts, including adolescents and young adults <24 years of age and shorter diabetes duration, and women using different methods of hormonal contraception are necessary. With the increasing availability of commercial AID options, it would also be of interest if similar experiences were being made by women using commercially developed AID systems.

In addition to our findings from this explorative study and qualitative analysis, future studies should focus on the analysis of diabetes device data from the AID systems in context with documentation of menstrual cycle data to provide a better understanding of the correlations that we found, identify patterns, investigate the efficacy and actual benefit of using workaround strategies compared to using an AID system with fixed settings, and set the stage for further automation tools and/or machine learning-supported AID for girls and women with menstrual cycles.

## Conclusions

Sex hormones are likely to directly or indirectly influence insulin requirements in women with and without T1D, although these correlations have so far not been sufficiently researched. In this study, we generated experience-based evidence of women of the #WeAreNotWaiting community which provides an overview on current challenges to address in future research and by developers of commercial and open-source AID. Due to the automation of insulin dosing and data tracking in AID, it should be possible to quantify recurring patterns in glycemic outcomes and insulin needs throughout the menstrual cycle.

Furthermore, the “workaround strategies” the women created provide useful information for potential further usability improvements and automation of control algorithms. As an example, the integration of menstrual cycle tracking data into AID systems could further improve safety and efficacy in users with menstrual cycles.

Last, awareness, existing scientific evidence, and professional guidance on the topic of female health in diabetes management are still insufficient. Therefore, we encourage an open dialogue on women’s health between women with T1D and health care professionals, and to consider cycle-related changes in insulin sensitivity when reviewing data and adjusting insulin dosage as part of their contacts. Moreover, further education and advocacy efforts are required to better inform PwD, health care professionals, and device manufacturers, and more research is required to better address the needs of women with T1D in therapeutic advances.

## Supplemental Material

sj-docx-1-dst-10.1177_19322968221080199 – Supplemental material for Variability of Glycemic Outcomes and Insulin Requirements Throughout the Menstrual Cycle: A Qualitative Study on Women With Type 1 Diabetes Using an Open-Source Automated Insulin Delivery System Click here for additional data file. Supplemental material, sj-docx-1-dst-10.1177_19322968221080199 for Variability of Glycemic Outcomes and Insulin Requirements Throughout the Menstrual Cycle: A Qualitative Study on Women With Type 1 Diabetes Using an Open-Source Automated Insulin Delivery System by Darius Mewes, Mandy Wäldchen, Christine Knoll, Klemens Raile and Katarina Braune in Journal of Diabetes Science and Technology
