# Supplementary material for: Variability of Glycemic Outcomes and Insulin Requirements Throughout the Menstrual Cycle: A Qualitative Study on Women With Type 1 Diabetes Using an Open-Source Automated Insulin Delivery System
Source: J Diabetes Sci Technol. 2022 Mar 7;17(5):1304–16. doi: 10.1177/19322968221080199 (PMC10563528; doi:10.1177/19322968221080199)
Supplement: sj-docx-1-dst-10.1177_19322968221080199 – Supplemental material for Variability of Glycemic Outcomes and Insulin Requirements Throughout the Menstrual Cycle: A Qualitative Study on Women With Type 1 Diabetes Using an Open-Source Automated Insulin Delivery System [file sj-docx-1-dst-10.1177_19322968221080199.docx]

# Supplementary Material

## Questionnaire for semi-structured interviews

**Demographics:**

- Age in years
- Biological sex
- Country of residence

**Diabetes history:**

- Year of T1D diagnosis
- Therapy regimen before starting open-source AID (e.g. MDI, insulin pump, use of CGM)
- Duration of using open-source AID
- Open-source AID type in use (OpenAPS, AndroidAPS, Loop, other)
- Insulin type
- Approximate % Time-in-Range with open-source AID
- Last HbA1c level (in % or mmol/mol)
- Comorbidities (e.g. celiac disease, thyroiditis)

**Gynaecological history:**

- Age at menarche and onset of menopause, if applicable
- Pregnancies and births (number of pregnancies, years, gestational age at birth, mode of delivery, occurrence of pregnancy complications, diabetes-associated complications of the child in the neonatal period)
- Known pre-existing female-health related conditions (e.g. PCOS)
- If applicable: contraceptive method(s)
- Current complaints (e.g. pain, infections, unfulfilled desire to have a child)

**Questions about the menstrual cycle:**

- Do you document your menstrual cycle (currently or in the past, is it documented regularly or irregularly)? What (electronic) tools do you use for this (e.g. apps, websites)?
- What information is recorded in the chosen method of cycle documentation? (e.g. Occurrence and duration of menstruation, physical symptoms, basal body temperature, ovulation, etc.)
- Are there any costs associated with the current documentation method? (e.g. app with costs)
- Which device is used for data entry (iPhone, Android phone, iPad, tablet, Computer etc.)?
- Approximate cycle length in days (and variability), regular or irregular cycle? Any known pattern or form of irregularities in the cycle?
- If yes, which ones? How do these influence your cycle?
- What physical and mental health changes do you experience during the cycle (e.g. mood swings, fatigue, pain, appetite, libido, skin, digestion, sleep, ability to exercise (due to pain, fatigue, etc.)?
- Do your glycaemic levels or insulin requirements change during the menstrual cycle? (e.g. between first half of the cycle, ovulation, second half of the cycle, before menstruation, during menstruation) How much does it change (estimated relative change in %) and in what period/with what rhythm?
- Are there any influences that additionally exacerbate the change of glycaemic levels and insulin requirements?
- Are regular changes in insulin requirements anticipated? Do you act proactively or reactively?
- If no clear correlation can be identified: Do you think a connection is likely? Likert scale (range) 1-5 (not likely - very likely); if yes/no: why (not)?
- Who provides support in using open-source AID in relation to therapy adjustment (e.g. diabetologist, gynaecologist, diabetes educator, family members, friends, peer-support groups)?
- If information is obtained from the online community: which and where exactly (e.g. specific social media channels or groups)?
- Do symptoms that occur during the cycle make it harder for you to carry out daily activities, commitments or obligations (e.g. work)?
- Do the glucose fluctuations during the cycle make it even more difficult for you to fulfill your obligations?
- How do you manage changes in insulin requirements? Do you manually change open-source AID settings (e.g. changes in ISF, basal rate profiles, overrides, temporary targets, preset profiles) or do you enter "fake carbs" to address the changes in insulin requirements? If so, which ones? Are these suitable for regular use or are there significant fluctuations between individual months?
- Would cycle-based automation of the closed-loop system be helpful? Likert scale 1-5 (not helpful - very helpful); if yes/no: why (not)?
- Are there any ideas and/or suggestions of your own as to which features closed-loop systems of the future should contain in order to better adapt diabetes therapy to the menstrual cycle or pregnancy?

**Further questions (optional):**

- Changes since menarche?
- Changes since menopause?
- Pregnancy: What did you notice regarding diabetes management occurring during and after pregnancy? Have there been any difficulties? How were these encountered? What worked well?
- Hormonal contraception: Which type (pill, contraceptive ring, IUD, which hormones)? Since when? Prescribed mainly for contraception or other complaints, e.g. skin complaints, menstrual pain, other pre-existing conditions, etc.? Were there any changes between phases with the use of hormonal contraceptives and those without?

**Supplementary Table 1:** Relative changes [%] in self-reported insulin requirements of female open-source AID users during different menstrual cycle phases.

| **#** | **Relative insulin needs [%]** | | |
| --- | --- | --- | --- |
|  | **Early follicular phase** | **Ovulation** | **Luteal phase** |
| **1** | 80 | 150 | 100 |
| **2** | 110 | 110 | 120 |
| **3** | 100 | 100 | 120 |
| **4** | 80 | 110 | 120 |
| **5** | 110 | 100 | 80 |
| **6** | 90 | 110 | 110 |
| **7** | 70 | 100 | 120 |
| **8** | 110 | 90 | 120 |
| **9** | 110 | 100 | 100 |
| **10** | 100 | 100 | 100 |
| **11** | 90 | 100 | 120 |
| **12** | 110 | 100 | 100 |
